# Post-Transplantation Cytomegalovirus Infection Interplays With the Development of Anastomotic Biliary Strictures After Liver Transplantation

**DOI:** 10.3389/ti.2022.10292

**Published:** 2022-06-02

**Authors:** Pauline Georges, Clémentine Clerc, Célia Turco, Vincent Di Martino, Brice Paquette, Anne Minello, Paul Calame, Joséphine Magnin, Lucine Vuitton, Delphine Weil-Verhoeven, Zaher Lakkis, Claire Vanlemmens, Marianne Latournerie, Bruno Heyd, Alexandre Doussot

**Affiliations:** ^1^ Department of Digestive Surgical Oncology –Liver Transplantation Unit, University Hospital of Besançon, Besancon, France; ^2^ Department of Hepatology, University Hospital of Dijon, Dijon, France; ^3^ Department of Hepatology, University Hospital of Besançon, Besancon, France; ^4^ Department of Radiology, University Hospital of Besançon, Besancon, France; ^5^ Department of Gastroenterology, University Hospital of Besançon, Besancon, France

**Keywords:** liver transplantation, anastomotic biliary complications, biliary reconstruction, CMV infection, primoinfection

## Abstract

**Background:** Anastomotic biliary stricture (ABS) remains the most frequent complication after liver transplantation (LT). This study aimed to identify new anastomotic biliary stricture risk factors, with a specific focus on postoperative events. Additionally, ABS management and impact on patient and graft survival were assessed.

**Methods:** All consecutive patients who underwent LT with duct-to-duct anastomosis between 2010 and 2019 were included. All patients who died within 90 days after LT due to non-ABS-related causes were excluded.

**Results:** Among 240 patients, 65 (27.1%) developed ABS after a median time of 142 days (range, 13–1265). Median follow-up was 49 months (7–126). Upon multivariable analysis, donor BMI (OR=0.509, *p* = 0.037), post-LT CMV primoinfection (OR = 5.244, *p* < 0.001) or reactivation (OR = 2.421, *p* = 0.015) and the occurrence of post-LT anastomotic biliary fistula (OR = 2.691, *p* = 0.021) were associated with ABS. Anastomotic technical difficulty did not independently impact the risk of ABS (OR = 1.923, *p* = 0.051). First-line ABS treatment was systematically endoscopic (100%), and required a median of 2 (range, 1–11) procedures per patient. Repeat LT was not required in patients developing ABS. The occurrence of ABS was not associated with overall patient survival (*p* = 0.912) nor graft survival (*p* = 0.521).

**Conclusion:** The risk of developing ABS after LT seems driven by the occurrence of postoperative events such as CMV infection and anastomotic fistula. In this regard, the role of CMV prophylaxis warrants further investigations.

## Graphical Abstract

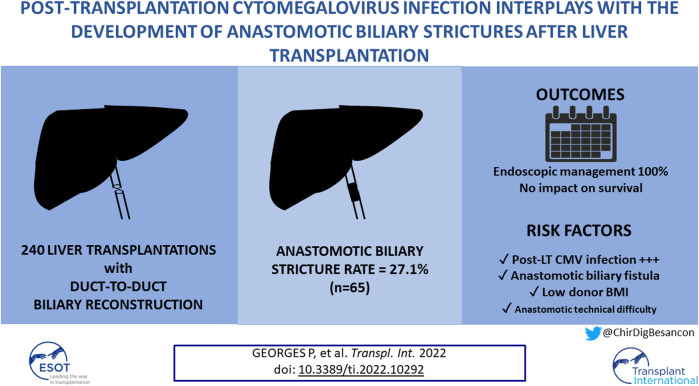

## Introduction

Although advances in organ preservation, immunosuppression, and surgical techniques have improved outcomes after liver transplantation (LT), biliary complications remain the most frequent cause of morbidity after LT (1). Biliary complications are conventionally classified as anastomotic biliary strictures (ABS), non-anastomotic biliary strictures, and anastomotic biliary fistula. Among these, ABS generally occurs within 1 year after transplantation and remains the most frequent biliary complication, accounting for 15.1%–35% of complications (2–4). Yet the physiopathology of ABS remains unclear. Due to the vulnerable vascularization of extrahepatic bile ducts, technical issues and local ischemia are risk factors classically reported in the literature (5, 6). Additionally, the use of the Model for End-stage Liver Disease (MELD) score for organ allocation and the expansion to extended criteria donor (ECD) have been associated with the risk of ABS (7, 8). Overall, risk factors are multiple as they are related to recipient and donor characteristics and transplantation techniques. Consequently, reported risk factors are highly variable and conflict between existing series (9, 7, 10, 11). Such a heterogeneity across the literature is explained in part by the lack of consensus on an ABS definition and the heterogeneity of included patients in terms of recipient severity, graft type, biliary reconstruction techniques, and biliary stricture types (e.g., anastomotic or not), among other potential risk factors. Notably, the increasing use of ECD-focused research on graft optimization to improve outcomes, along with other factors such as postoperative events, might interplay with the occurrence of ABS.

This study aimed to identify preoperative, intraoperative, and postoperative risk factors of ABS after deceased donor liver transplantation. Additionally, ABS management and impact on patient and graft outcomes were evaluated.

## Methods

### Study Population

All consecutive patients who underwent LT with duct-to-duct anastomosis between January 2010 and December 2019 were considered for inclusion. All patients who underwent a bilio-enteric reconstruction and those lost in follow-up or requiring early retransplantation after LT were excluded. Additionally, to avoid competing risks of early mortality due to causes other than ABS, patients who died within 90 days post-LT owing to ABS-unrelated causes were excluded. Recipients were divided into two groups based on the occurrence of ABS or not.

### Data Collection

Data were retrieved from electronic medical records and from the prospectively maintained CRISTAL on-line data base from the Biomedicine Agency, approved by the French Data Protection Authority (Commission Nationale de l’Informatique et des Libertés) (Decision n◦96-025 of March 19 1996). The following data were collected: recipients’ characteristics at the time of transplantation, donors’ characteristics, intraoperative data, and postoperative outcomes. Extended criteria donors were those older than 75 years, or with confirmed steatosis >30%. All postoperative complications occurring within 90 days after surgery were collected and graded according to the Dindo–Clavien classification. In patients with multiple complications, the highest grade was retained for analysis. Regarding anastomotic biliary complications other than ABS, anastomotic biliary fistula was defined according to the ISGLS definition (12). Early allograft dysfunction was defined according to the current definition (13). Early rejection corresponded to a histological diagnosis defined upon Banff criteria within the first 3 months after LT (14). Medical complications including extra-abdominal infection, CMV infection, or reactivation and acute kidney injury were also collected.

### Liver Graft Procurement and Transplantation

All grafts were procured from a brain-dead donor using the *en bloc* technique (15) unless the pancreas was simultaneously procured for organ transplantation. Full dissection of the graft hepatic pedicle was next carried out on a hypothermic back table using an ice basin filled with cold preservation solution. Care was taken not to dissect above the gastroduodenal artery to prevent any proper hepatic artery lesion. Similarly, cholecystectomy was performed at this stage and the common hepatic duct was bluntly dissected and divided as low as possible after pancreatic head removal to avoid any injury or devascularization.

Regarding biliary reconstruction after vascular implantation, duct-to-duct anastomosis was performed using a 6/0 or 7/0 polyglyconate long-term monofilament absorbable sutures (Maxon™, Covidien, Medtronic, Watford, United Kingdom). Both graft and recipient bile duct were trimmed to length to ensure a tension-free anastomosis between two appropriately vascularized ducts. End-to-end anastomosis was then fashioned using a posterior running suture and anterior interrupted suture or two posterior and anterior running sutures, systematically knotted on the outside. In case of anticipated anastomotic difficulty, an anterior ductoplasty technique or a T-tube insertion could be used at the discretion of the transplant surgeon. Anastomotic difficulty was anticipated when the graft and/or the recipient bile duct diameter was smaller than 5 mm or when a donor-recipient duct size mismatch larger than 4 mm was present.

### Postoperative Management

Systematic Doppler ultrasounds were performed daily from postoperative day one to five, then once a week to detect any vascular complication. Pre-transplantation screening of donors and recipients for CMV serological status (IgG) defined the strategy employed for the prevention of CMV reactivation or primary infection. A 6-month CMV prophylaxis was routinely given to “high-risk” recipients defined as seronegative recipients receiving a graft from a seropositive donor. The pre-emptive strategy was applied to other patients with routine determination of CMV viremia by sensitive assay (molecular diagnosis). Of note, in this situation, CMV antigenemia assay and qPCR were weekly checked from LT to patient discharge, then once a month for the first year. In case of positive CMV viremia, whether symptomatic or not, CMV therapy was systematically initiated to prevent progression to clinical disease.

The diagnosis of ABS was suspected on the presence of a size discrepancy at the site of the bilio-biliary anastomosis with or without upstream bile duct dilatation on imaging (ultrasound, cholangiography, CT scan, or MR-cholangiography). This had to be associated with a concomitant cholestasis and/or an elevated serum bilirubin after excluding other cholestasis causes such as graft rejection and viral reactivation. Each patient with suspected ABS underwent an endoscopic retro-grade cholangiopancreatography (ERCP) to confirm ABS. In case of ABS confirmation, a plastic or self-expandable metallic stent was placed at the discretion of the endoscopic team. Endoscopic stent replacement was repeated each 4–6 months until ABS clearance.

### Statistical Analysis

The χ^2^ test or Fischer exact test was used for analysis of categorical variables, as appropriate. Continuous variables with a normal distribution were presented as mean (standard deviation) and non-normally distributed variables as median (range); t test and Mann–Whitney U test were used for statistical analysis. All perioperative variables associated with the occurrence of ABS in univariable analysis (*p* < 0.100) were included in a binary logistic regression model to identify independent risk factors of ABS. Backward selection was used, with a 0⋅1 cut-off for entry into the model. In case of collinearity between variables, only the most relevant variable was included in the model. Regarding postoperative variables, given the potential time-dependent relationship between the occurrences of postoperative events, only those occurring before the occurrence of ABS were deemed of interest and were retained in multivariable analysis. Performance of the multivariable model was assessed in terms of discrimination, expressed as the area under the curve (AUC) ± standard error (SE). Additionally, overall and graft survival estimates were calculated using the Kaplan–Meier method. OS and graft survival corresponded to the interval between LT and the date of last follow-up or death and between LT and date of graft failure. Survival differences between patients who did and did not experience ABS were compared using the log rank test. All *p* values were based on two-tailed statistical analysis and *p* < 0⋅050 was considered to indicate statistical significance. Analyses were performed with SPSS^®^ software, version 27.0 for Windows^®^ (IBM, Armonk, New York, United States). The present study complied with the STROBE Guidelines (16).

## Results

### Population

**FIGURE 1:**
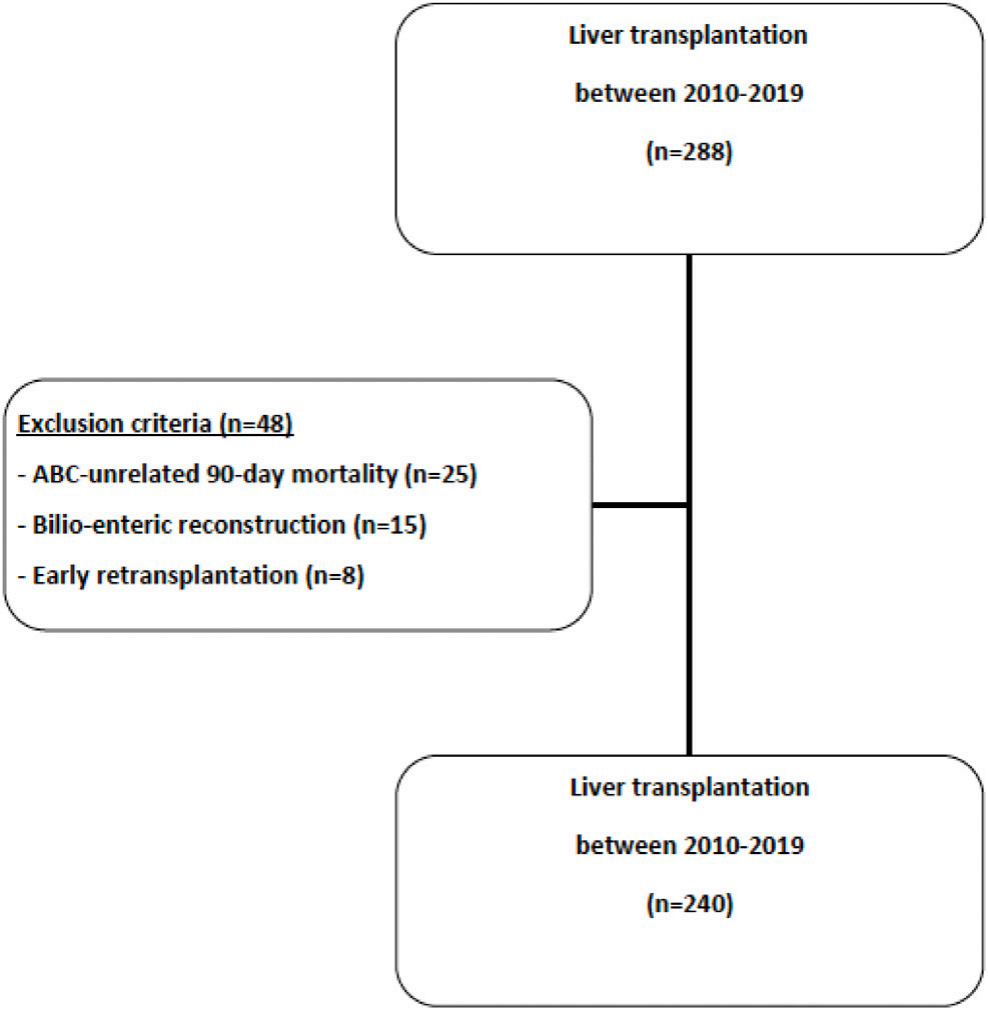
Study flowchart.

Over the study period, 288 LT were performed, of which 48 LT were excluded (ABS-unrelated 90-day mortality in patients with bilio-biliary reconstruction, *n* = 25; bilio-enteric reconstruction, *n* = 18; early retransplantation, *n* = 5) and 240 LT were included (Figure 1). Recipients’ characteristics are listed in Table 1. Mean recipient age and mean MELD score were respectively 55.7 years old and 21.2 at the time of transplantation. Donors’ characteristics are listed in Table 2. Mean donor age was 57.6 years old. Most grafts (90.4%) were allocated according to the standard national liver score system. ECD was used in 141 LT (58.8%). Main causes for brain death were stroke (58.2%) and trauma (24.6%) and nearly one third (28.7%) of donors presented cardiac arrest. Pre-transplantation donors’ and recipients’ serological CMV status are described in Table 2.

**TABLE 1:** Characteristics of recipients at transplantation (*n* = 240).

	Overall (*n* = 240)	ABS+ (*n* = 65)	ABS- (*n* = 175)	*P*
Age, years	55.7 (10.4)	56.7 (10)	55.1 (10)	0.090
Gender				0.711
Male	173 (72.1%)	48 (73.8%)	125 (71.4%)	
Female	67 (27.9%)	17 (26.2%)	50 (28.6%)	
Body mass index, kg/m^2^	26.4 (4.8)	26 (4)	26 (5)	0.605
Preoperative recipient morbidity and acuity				
Hypertension	79 (32.9%)	23 (35.4%)	56 (32%)	0.620
Type 2 diabetes	69 (28.7%)	17 (26.2%)	52 (29.7%)	0.588
Active smoking	67 (27.9%)	16 (24.6%)	51 (29.1%)	0.492
Mechanical ventilation (24 h pre- LT)	9 (3.8%)	2 (3.1%)	7 (4%)	0.738
Preoperative organ failure	7 (2.9%)	—	7 (4%)	0.204
MELD score	21.2 (9.8)	21.7 (10)	21 (10)	0.656
Child-Pugh score				0.170
A	65 (27.1%)	13 (20%)	52 (29.7%)	
B	41 (17.1%)	15 (23.1%)	26 (14.9%)	
C	134 (55.8%)	37 (56.9%)	97 (55.4%)	
Main indication for LT				0.186
Cirrhosis	138 (57.5%)	43 (66.2%)	95 (54.3%)	0.237
Hepatocellular carcinoma	59 (24.6%)	12 (18.5%)	47 (26.9%)	0.108
Acute liver failure	22 (9.2%)	7 (10.8%)	15 (8.6%)	0.600
Other	17 (7.1%)	3 (4.6%)	14 (8%)	0.571
Repeat LT	4 (1.7%)		4 (2.3%)	0.577
Previous upper GI surgery	36 (15%)	12 (18.5%)	24 (13.7%)	0.360
Waiting-list time, months	3 (0–56)	2 (0–38)	3 (0–56)	0.415

Numbers are expressed as mean (standard deviation), unless otherwise specified LT, liver transplantation; MELD, Model for End-stage Liver Disease.

**TABLE 2:** Characteristics of donors (*n* = 240).

	Overall (*n* = 240)	ABS+ (*n* = 65)	ABS- (*n* = 175)	*P*
Age, years	57.6 (18.5)	58.8 (20)	57.2 (18)	0.325
Gender				0.827
Male	132 (55%)	35 (53.8%)	97 (55.4%)	
Female	108 (45%)	30 (46.2%)	78 (44.6%)	
Body mass index, kg/m^2^	25.6 (4.9)	24.5 (4.3)	26.1 (5.1)	0.015
Graft allocation type				0.796
Acute	15 (6.3%)	5 (7.7%)	10 (5.7%)	
Standard	217 (90.4%)	57 (87.7%)	160 (91.4%)	
Hors-tour	6 (2.5%)	2 (3.1%)	4 (2.3%)	
DCD	2 (0.8%)	1 (1.5%)	1 (0.6%)	
Cardiac arrest	69 (28.7%)	19 (29.2%)	50 (28.6%)	0.920
Down time, minutes	25 (18.9)	30.4 (29.6)	22.9 (12.3)	0.891
Cause of death				0.512
Stroke	137 (58.2%)	37 (56.9%)	100 (57.1%)	
Trauma	59 (24.6%)	18 (27.8%)	41 (23.4%)	
Anoxia	37 (15.4%)	7 (10.8%)	30 (17.1%)	
Other	7 (2.9%)	3 (4.6%)	4 (2.3%)	
Donor morbidity				
Active smoking	94 (39.2%)	25 (38.5%)	69 (39.4%)	0.892
Alcohol intoxication	27 (11.3%)	3 (4.6%)	24 (13.7)	0.064
Hypertension	85 (35.4%)	21 (32.3%)	64 (36.6%)	0.539
Diabetes	34 (14.2%)	9 (13.8%)	25 (14.3%)	0.931
Cardiovascular disease	39 (16.3%)	11 (16.9%)	28 (16%)	0.863
Donor-recipient ABO matching	232 (96.7%)	62 (95.4%)	170 (97.1%)	0.530
Extended criteria donor	141 (58.8%)	103 (58.9%)	38 (58.5%)	0.956
Donor-recipient CMV status				0.032
Donor- Recipient-	50 (20.8%)	7 (10.8%)	43 (24.6%)	Reference
Donor + Recipient-	72 (30%)	24 (36.9%)	48 (27.4%)	0.009
Donor + Recipient+	62 (25.8%)	18 (27.7%)	44 (25.1%)	0.044
Donor- Recipient+	56 (23.3%)	16 (24.6%)	40 (22.9%)	0.046

Numbers are expressed as mean (standard deviation), unless otherwise specified CMV, Cytomegalovirus; DCD, Donor after Circulatory Death; ECD, Extended criteria donor.

Intraoperative data are reported in Table 3. Mean cold ischemia duration was 533.2 min. Anastomotic technical difficulty as defined above was encountered in 81 LT (33.8%). Both biliary ductoplasty (*n* = 45; 18.8%) and T-tube placement (*n* = 46; 19.2%) were not performed routinely. Owing to significant bleeding (*n* = 4) or failure to fascial closure (*n* = 3), an open abdomen approach with negative wound therapy was adopted at the end of LT in seven patients (2.9%), of which one had a delayed biliary reconstruction.

**TABLE 3:** Intraoperative data (*n* = 240).

	Overall (*n* = 240)	ABS+ (*n* = 65)	ABS- (*n* = 175)	*P*
Operative time, minutes	343 (78.4)	328 (69)	349 (81)	0.185
Estimated blood loss, l	3.4 (3.1)	3.3 (3.3)	3.4 (3)	0.221
Cold ischemia duration, minutes	533 (114)	536 (120)	532 (112)	0.959
Intraoperative red pack transfusion	194 (80.8%)	50 (76.9%)	144 (82.3%)	0.348
Intraoperative fresh frozen plasma	185 (77.1%)	48 (73.8%)	137 (78.3%)	0.467
Intraoperative platelets transfusion	120 (50%)	32 (49.2%)	88 (50.3%)	0.885
Temporary portocaval shunt	67 (27.9%)	20 (30.8%)	47 (27.8%)	0.654
Arterial reconstruction				0.809
One anastomosis	216 (90%)	59 (90.8%)	157 (89.7%)	
Two anastomoses	24 (10%)	6 (9.2%)	18 (10.3%)	
Caval replacement	4 (1.7%)	2 (3.1%)	2 (1.1%)	0.301
Portal thrombectomy	25 (10.4%)	3 (6.7%)	22 (15.4%)	0.206
Anastomotic technical difficulty	81 (33.8%)	31 (47.7%)	50 (28.6%)	0.005
Biliary ductoplasty	45 (18.8%)	11 (25%)	34 (24.8%)	0.981
T-tube use	46 (19.2%)	9 (13.8%)	37 (21.1%)	0.268
Delayed biliary reconstruction	1 (0.4%)	—	1 (0.6%)	>0.999
Open abdomen	7 (2.9%)	1 (1.5%)	6 (3.4%)	0.678

Numbers are expressed as mean (standard deviation), unless otherwise specified.

### Postoperative Outcomes

Among all transplanted patients with bilio-biliary reconstruction over the study period (*n* = 288), the 90-day mortality rate was 10.4% (*n* = 25). Causes of 90-day mortality are listed in Supplementary Table S1. No patient died due to an anastomotic biliary fistula or stricture after a bilio-biliary reconstruction. Among excluded patients, one patient with bilio-enteric reconstruction died of multiorgan failure due to an anastomotic biliary fistula.

Surgical reintervention within 90 days after LT was needed in 34 patients (14.2%), mostly for hemorrhage (*n* = 24), anastomotic biliary fistula (*n* = 8), arterial complication (*n* = 3), large for size syndrome (*n* = 2), and portal vein thrombosis (*n* = 1). Postoperative outcomes are displayed in Table 4.

**TABLE 4:** Postoperative complications after LT (*n* = 240).

	Overall (*n* = 240)	ABS+ (*n* = 65)	ABS- (*n* = 175)	*P*
Intensive care unit stay, days	8.4 (11)	7.5 (6)	8.7 (12)	0.918
Early allograft dysfunction	77 (33.5%)	18 (27.7%)	59 (33.7%)	0.333
Biliary complications	89 (37.1%)			
Anastomotic stenosis	65 (27.1%)	65 (100%)	—	—
Non anastomotic stenosis	20 (8.3%)	4 (6.2%)	16 (9.1%)	0.603
Anastomotic fistula	31 (12.9%)	16 (24.6%)	15 (8.6%)	0.001
Arterial complications	26 (10.9%)	9 (13.8%)	17 (9.7%)	0.360
Portal vein thrombosis	4 (1.7%)	1 (1.5%)	3 (1.7%)	>0.999
Intraabdominal infection	38 (15.8%)	6 (9.2%)	32 (18.3%)	0.111
CMV infection				<0.001
None	119 (49.6%)	42 (17.5%)	79 (32.9%)	
Primoinfection	42 (17.5%)	20 (30.8%)	22 (12.6%)	
Reactivation	79 (32.9%)	26 (40%)	53 (30.3%)	
Acute kidney injury	164 (68.3%)	46 (70.8%)	118 (67.4%)	0.621
Reoperation	34 (14.2%)	12 (18.5%)	22 (12.6%)	0.245
Early rejection	46 (19.2%)	11 (16.9%)	35 (20%)	0.713

Numbers are expressed as mean (standard deviation), unless otherwise specified CMV, cytomegalovirus.

### Incidence and Risk Factors of Anastomotic Biliary Strictures

Median follow-up was 49 months (range, 7–126). Overall ABS rate was 27.1% (*n* = 65), of which 20 (33.8%) developed within 90 days after LT. Median time to ABS diagnosis was 142 days (range, 13–1,265).

**FIGURE 2:**
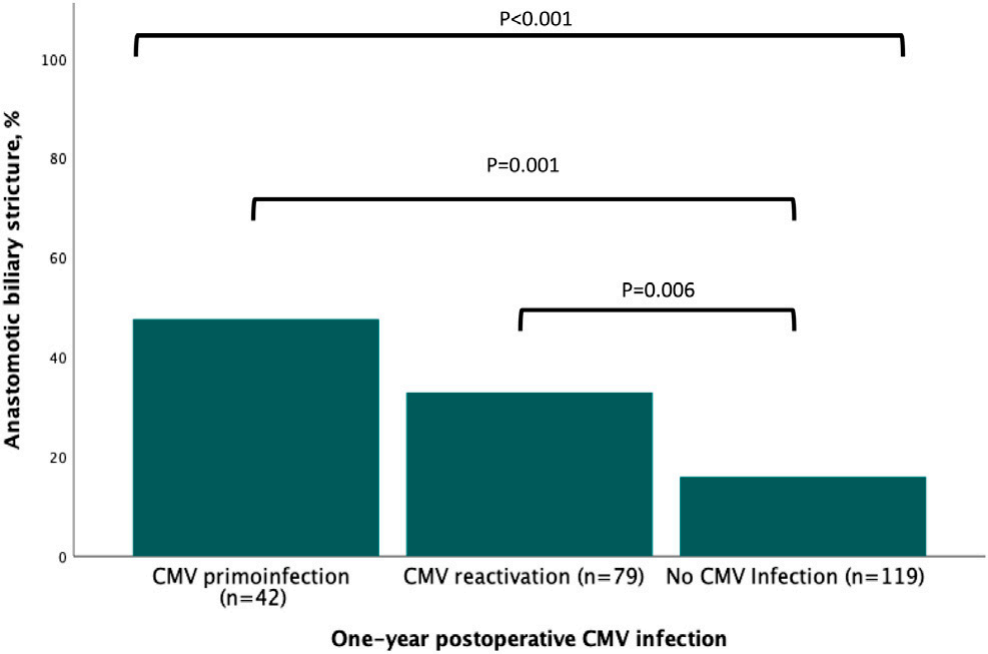
Association between the occurrence of postoperative infection and anastomotic biliary stricture.

Upon univariable analysis, ABS risk factors related to donor, recipient, intraoperative characteristics, and postoperative outcomes are listed in Tables 1–4. Recipient age (*p* = 0.090), donor BMI (*p* = 0.015), and donors’ and recipients’ serological CMV status (*p* = 0.030) were statistically associated with ABS. Intraoperatively, the existence of anastomotic technical difficulty was associated with ABS (*p* = 0.005). Regarding postoperative events, both the occurrence of anastomotic biliary fistula (*p* < 0.001) and a CMV infection (*p* < 0.001, Figure 2) were associated with ABS.

Upon multivariable analysis, elevated donor BMI (OR = 0.509, CI95% 0.270–0.959; *p* = 0.037), postoperative CMV primoinfection (OR = 5.244, CI95% 2.281–12.054; *p* < 0.001) or CMV reactivation (OR = 2.421, CI95% 1.192–4.920; *p* = 0.015) and the occurrence of anastomotic biliary fistula (OR = 2.691, CI95% 1.162–6.233; *p* = 0.021) were independently associated with ABS, although anastomotic technical difficulty did not reach a statistically significant association (Table 5). Discrimination of the estimated risks from the multivariable analysis was deemed acceptable (AUC, 0.740; SE, 0.035, *p* < 0.001).

**TABLE 5:** Risk factors for anastomotic biliary strictures in multivariable analysis (*n* = 240).

	OR	CI95%	*P*
Recipient and donor characteristics			
Recipient age	1.002	0.972–1.033	0.906
Donor BMI >25 kg/m^2^	0.509	0.270–0.959	0.037
Extended criteria donor	0.972	0.509–1.856	0.932
Intraoperative characteristics			
Anastomotic technical difficulty	1.923	0.996–3.712	0.051
Postoperative complications			
Anastomotic biliary fistula	2.691	1.162–6.233	0.021
CMV infection			<0.001
None	Reference		
Primoinfection	5.244	2.281–12.054	<0.001
Reactivation	2.421	1.192–4.920	0.015

Numbers are expressed as mean (standard deviation), unless otherwise specified BMI, body mass index; CMV, cytomegalovirus.

### Interaction Between Anastomotic Biliary Strictures and Statistically Significant Postoperative Events

Among 65 patients who developed ABS, 46 (70%) experienced postoperative CMV infection, of which eight (17%) were under CMV prophylaxis. Among these 46 patients, 34 patients (75%) developed first postoperative CMV infection before experiencing ABS diagnosed after a median of 172 days after LT (range, 18–1,222). In contrast, 12 patients (25%) developed first ABS with a median interval after LT of 131 days (range, 30–447) before presenting CMV infection.

Similarly, 31 (12.9%) patients experienced anastomotic biliary fistula including three grade A (10%) and 22 grade B (71%) all managed endoscopically, either alone (*n* = 15) or combined with a percutaneous drainage (*n* = 7). The remaining six patients (19%) were deemed grade C as they required reoperation. Of them, 15 (48%) developed subsequent ABS after a median interval between anastomotic biliary fistula and stricture of 41 days (range, 5–239).

### Management of ABS and Impact on Long-Term Outcomes

First-line ABS treatment was systematically endoscopic (100%), requiring a median treatment duration of 252 days (range, 133–912) for a median number of two (range, 1–11) procedures per patient. Twelve patients (18.5%) eventually developed ABS recurrence, of which nine were managed endoscopically, two percutaneously, and one surgically. Repeat LT was not required due to ABS but was performed in 11 patients owing to ischemic cholangitis (*n* = 5), acute hepatic artery thrombosis (*n* = 3), and chronic rejection (*n* = 3).

**FIGURE 3:**
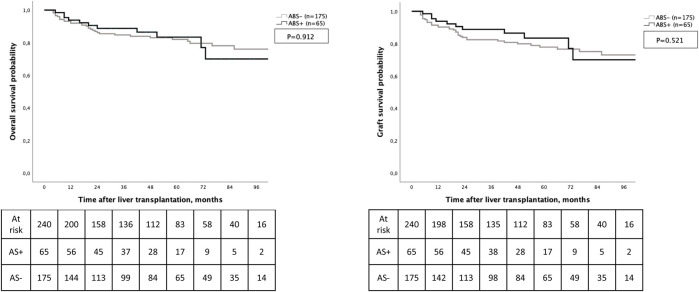
Overall survival and graft survival according to the development of anastomotic biliary stricture.

Regarding long-term outcomes, 1-year and 5-year overall survival and graft survival rates were respectively of 93%, 72% and 92%, 71%. The occurrence of ABS was not associated with OS (*p* = 0.912) and graft survival (*p* = 0.521) (Figure 3).

## Discussion

In the current study, the ABS rate was 27.1% and reported rates classically range from 15 to 35% (2–4). Such variations in the literature are likely related to multiple factors. Mostly, no consensus definition and monitoring guidelines are available. This results in heterogeneous detection rates, causing unbiased comparisons between existing series in the literature. In the current series, 10 patients (15%) underwent ERCP with biliary stenting while no upstream biliary dilatation was found at MR-cholangiography and ERCP (17). While this remains difficult to ascertain that these patients had definitive bona fide ABS, given the absence of consensus definition, such clinical situations might not have been considered as ABS at other institutions.

The occurrence of ABS is often attributed to the use of marginal donors. In the current study, liver graft from ECD was used in nearly 60% of LT and was not associated with the occurrence of ABS. Instead, three independent risk factors have been identified. Although the protective role of high donor BMI on the occurrence of ABS remains difficult to discuss, Jarlot-Gas et al. recently reported the same association (3). One could hypothesize that high donor BMI is related to large graft bile duct, resulting in less difficult biliary reconstruction. More importantly, the predominant role of postoperative events on the development of ABS has to be highlighted. Regarding the association between the occurrence of anastomotic biliary fistula and the development of ABS, this relationship has been previously shown in various series (3, 18, 19). Anastomotic biliary fistula would indeed cause local inflammation, eventually leading to local fibrosis at the site of healing, resulting in ABS.

More interestingly, the occurrence of postoperative CMV infection was independently associated with the risk of ABS. Upon univariable analysis, pre-transplantation donors’ and recipients’ serological CMV status was associated with an increased risk of ABS. This suggested the propensity of patients at risk of postoperative CMV infection to develop ABS. This was confirmed upon multivariable analysis as the occurrence of CMV primo-infection or reactivation was independently associated with an increased risk of developing ABS. Overall, the majority of patients who developed ABS presented CMV infection in their postoperative course. In most cases, CMV infection preceded ABS diagnosis and was virally reactivated. While the relevance of CMV detection and prevention after LT has been largely established, the association between CMV infection and ABS development remains unclear (20–22). Among the large existing body of literature focused on ABS risk factors, very few series have found similar findings (23–25). The current study was not designed to investigate mechanisms of CMV on ABS occurrence, but one can hypothesize that destruction of vascular endothelial cells due to CMV infection might lead to arterial thrombosis, resulting in biliary ischemia (26). Additionally, it has been shown that CMV can be latent in epithelial cells and be shed in bodily fluids (27). Notably, CMV DNA has been found to be more prevalent in biliary fluid than in liver biopsy or blood serum after LT (28). Gotthardt et al. reported that the presence of CMV DNA in the biliary tract after LT was significantly associated with the development of biliary stricture (29). However, the presence of CMV DNA in bile was significantly associated with non-anastomotic biliary stricture instead of ABS. Future investigations are consequently needed to further understand mechanism and develop prevention and treatment strategies (30).

In addition to preoperative characteristics and postoperative events, intraoperative data were also investigated. Upon univariable analysis, only the anastomotic technical difficulty defined as bile duct diameter smaller than 5 mm or donor-recipient duct size mismatch larger than 4 mm was found associated with an increased risk of ABS. Yet, in the current study, this failed to reach statistical significance upon multivariable analysis. However, a tiny duct size has been already reported as an ABS risk factor in multiple series including one randomized trial (25, 31). In order to overcome the technical difficulty, using ductoplasty techniques or T-tube insertion was at the discretion of the surgeon. As previously shown, none of these technical refinements were associated with a reduced ABS rate (32–34).

Regarding ABS management and impact on survival, the current study confirmed findings from a large body of the previously published literature. First, ABS endoscopic management was effective in most cases as a first-line approach with a recurrence rate around 20% (3, 35–38). Further, even in case of ABS recurrence, repeat endoscopic treatment allowed a successful treatment in most cases, thereby obviating the need for percutaneous transhepatic or surgical treatment (35, 39). Second, the occurrence of ABS did not negatively impact long-term survival. This observation is in line with other series (3, 40, 41). Yet, a recent large study showed the negative impact of early anastomotic biliary complication occurring within the first 3 months after LT (42). Nevertheless, such contrasting results from this series among others must be interpreted cautiously (43, 44). These series are indeed heterogenous in terms of study period, biliary complications timing, e.g. early or late, types e.g., anastomotic or not, definitions, and management. In contrast, the current single center cohort, despite spanning over 9 years, was focused on ABS after LT with duct-to-duct reconstruction using total liver grafts from brain dead donors and provided a certain homogeneity in terms of management with all patients following a standardized management pathway whether regarding perioperative monitoring or intraoperative techniques.

In addition to its retrospective nature limiting any causality analysis, some limitations of the present study have to be discussed. First, when ABS is suspected, graft rejection is also classically suspected. This might lead to intensified immunosuppression or cessation of CMV prophylaxis, thereby contributing to a higher risk of CMV infection. It may be difficult to disentangle cause and consequence in this setting. Second, the study period may implicate time lead bias, especially considering potential changes in organ preservation protocols and surgical techniques over time. However, there was no significant change within the last decades. Third, intraoperative data such as reperfusion syndrome, arterial ischemia duration, or the use of vasopressive drugs were not available in the data set. Such data might be associated with the development of ABS. Fourth, the occurrence of postoperative CMV infection was independently associated with the risk of ABS. However, quantitative data on CMV viral load in the blood was lacking for some patients. Further, no data was available on the presence of CMV in the bile, whether at the time of LT or later. Finally, external validation would be of value to confirm the impact of postoperative CMV infection as well as performances of the multivariable model.

In conclusion, the risk of developing ABS after LT is multifactorial but seemed mostly driven by the occurrence of postoperative events such as CMV infection, especially primoinfection and anastomotic biliary fistula. Generally managed endoscopically, ABS did not seem to impact survival after LT.

## Data Availability

The raw data supporting the conclusion of this article will be made available by the authors, without undue reservation.

## Abbreviations

ABS, anastomotic biliary stricture; BMI, body mass index; CMV, cytomegalovirus; DNA, desoxyribonucleic acid; ECD, extended criteria donor; ERCP, endoscopic retrograde cholangio pancreatography; ISGLS, international study group for liver surgery; LT, liver transplantation; MELD, model for end stage liver disease; PCR, polymerase chain reaction.
