# Supplementary material for: Post-Transplantation Cytomegalovirus Infection Interplays With the Development of Anastomotic Biliary Strictures After Liver Transplantation
Source: Transpl Int. 2022 Jun 2;35:10292. doi: 10.3389/ti.2022.10292 (PMC9200969; doi:10.3389/ti.2022.10292)
Supplement: Supplementary file 1 [file Table1.docx]

| Supplementary Table 1. Causes of 90-day mortality in patients with bilio-biliary reconstruction (n=25) | | |
| --- | --- | --- |
|  | |  |
| Sepsis  - Extraabdominal  - Intraabdominal | 7  5 | |
| Hepatic artery thrombosis | 5 | |
| Portal vein thrombosis | 4 | |
| Haemorrhage | 1 | |
| Primary non function | 1 | |
| Others |  | |
| - Myocardial infarction  - DRESS syndrome | 1  1 | |
| DRESS; drug rash with eosinophilia and systemic symptoms | | |
